# Rapid Estimation of Parameters for Gelatinization of Waxy Corn Starch

**DOI:** 10.3390/foods8110556

**Published:** 2019-11-06

**Authors:** Robert Bertrand, William Holmes, Cory Orgeron, Carl McIntyre, Rafael Hernandez, Emmanuel D. Revellame

**Affiliations:** 1 Department of Chemical Engineering, University of Louisiana at Lafayette, Lafayette, LA 70504, USA; 2 The Energy Institute of Louisiana, University of Louisiana at Lafayette, Lafayette, LA 70504, USA; 3 Department of Industrial Technology, University of Louisiana at Lafayette, Lafayette, LA 70504, USA

**Keywords:** excess water condition, gelatinization temperature, G endotherm, gelatinization time

## Abstract

Starch gelatinization is an important process due to the prevalence of starch usage in industries such as cosmetics and food production. In this study, the gelatinization of waxy corn starch (WCS) was investigated with the goal of providing an option for the rapid determination of starch gelatinization characteristics. The procedure used in the study was solely based on differential scanning calorimetry (DSC), which is an established technique for the determination of thermal characteristics of starches. A sequence of experiments was conducted to determine the excess water condition, an estimate of the minimum gelatinization temperature, and gelatinization time. These parameters were found to be ≥65 wt.% water, 75–85 °C, and 10 min, respectively. The estimation of the minimum gelatinization temperature was determined from the thermal properties of the WCS as obtained by DSC. The obtained parameters resulted in complete WCS gelatinization, and, thus, the sequence of procedures used in the study could possibly be used for rapid waxy starch evaluation.

## 1. Introduction

Starch is an important food polymer that is the major source of carbohydrate intake for the world’s population. The use of starch in food is nearly universal (e.g., as a thickener for soups, sauces, gravies, baby foods and pudding, as well as an ingredient for cakes, jelly confectioners, breads, noodles, canned and frozen foods). In addition to these applications in the food industry, starches have uses that extend to sizing and coating papers, textile sizing, drilling muds, adhesive formulations, biocomposites, copolymers, plasticizers, biomedical applications, and others [1,2,3]. Of the many varieties of crops grown for starch production, the three major crops are wheat, maize (corn), and rice. These three crops account for 94% of worldwide starch consumption [4]. For most starch applications, gelatinization, which is a phase transition of starch granules from an ordered to a disordered state during heating, is necessary [1]. In other words, gelatinization is a broad term given to the irreversible endothermic process of the heating or cooking of starch to solubilize it in water [5]. This process is primarily a function of starch (or water) concentration, gelatinization/cooking/treatment temperature, and to a lesser degree, treatment time. This process is generally short, according to Lund and Wirakartakusumah [6], who reported that rice starch gelatinization concludes within 2–3 min. Since this process is used extensively in the food industry, the rapid determination of gelatinization parameters is exceedingly useful in process development and improving process efficiency.

Studies [7,8,9] have indicated that starch gelatinization is more difficult to accomplish in the limited or low water condition (where starch is in excess) due to the unavailability of enough water to swell starch granules and access their internal structures for solubilization. This implies that from a process perspective, gelatinization should be accomplished in the excess water or high water conditions. However, too much water should also be avoided to minimize the energy needed to heat excessive and unnecessary amounts of water during the process. In the excess water condition, studies have indicated that the heat associated with gelatinization per unit mass of starch (also known as specific heat or enthalpy of gelatinization) is relatively constant [10,11,12]. This leads to a water content where the process transitions/shifts from the limited water condition to the excess water condition, which is also the minimum water content needed for the gelatinization process to easily proceed to completeness. This water content (“transitional water content”) is the ideal or optimum water content during starch gelatinization because it represents the lowest water content within the excess water condition.

In the excess water condition, gelatinization proceeds rapidly to a certain extent which depends on cooking temperature. Several studies [6,12,13,14,15] have indicated that for a given water content, there is a minimum temperature, below which complete starch gelatinization cannot be accomplished. The degree of gelatinization completion is designated as the terminal extent of gelatinization (TEG) [9], which, due to its linear relationship with the enthalpy of gelatinization (∆*H*) [6], can be calculated using the equation:

*TEG* = (∆*H*_n_ − ∆*H*_r_)/∆*H*_e_ (1)

where ∆*H*_n_ is the specific gelatinization enthalpy, ∆*H*_r_ is the residual specific gelatinization energy after the treatment of the starch/water mixture, and ∆*H*_e_ is the ∆*H*_n_ at the excess water condition. ∆*H*_e_ is the maximum ∆*H* that represent 100% gelatinization of a given starch, while the difference between ∆*H*_n_ and ∆*H*_r_ can be thought of as the enthalpy used for the partial gelatinization during treatment.

The TEG or degree of gelatinization can be measured using various techniques including differential scanning calorimetry (DSC) [6,9,12,14,16], enzymatic digestion [13,17], electrical conductivity [18], and dough rheology [19]. Among these techniques, DSC has been the most utilized method because it provides the properties of the endothermic gelatinization process, such as enthalpies (that can be used for TEG calculations), the characteristic temperatures of endotherms, and insights into the transitional water content.

Most studies on starch gelatinization have been focused on the elucidation of phase transition mechanisms. As such, they have typically been limited to studies on various water contents and temperatures at specified single treatment times. These types of studies are tedious and limited by time and resources that are not always available. With the continuous development on plant breeding, starch isolation, and modifications [3], a rapid method of determining gelatinization parameters will greatly aid in process development and optimization. Thus, this study was conducted using a simple procedure to investigate the gelatinization behavior of waxy cornstarch (WCS) using DSC. In particular, this study aimed to determine the transitional water content, an estimate of the optimal (or minimum) cooking temperature in the excess water condition, and the minimum time required at this temperature to fully gelatinize WCS. WCS applications within the excess water condition were specifically targeted for this study.

## 2. Materials and Methods

The deionized water used in all experiments was filtered and deionized by a Millipore (Burlington, MA, USA) Milli-Di filtration and deionization system. The WCS (Waxy #1) was purchased from Tate & Lyle PLC (Kingsway, London, UK) and had the characteristics presented in Table 1, as provided by the manufacturer. The N_2_ gas for DSC operation was obtained from Red Ball Oxygen (Alexandria, LA, USA).

All experiments were carried out on a SDT 2960 DSC/TGA (Thermogravimetric analysis) (TA Instruments, New Castle, DE, USA), which was calibrated using company-supplied sapphire calibrant at 40–150 °C. The TA Universal Analysis software was used to analyze the resulting DSC traces and curves. Hermetically sealed aluminum pans (4 × 6 mm) with flat bottoms were used to contain samples. A Cahn C-30 microbalance with a precision of 0.001 mg was used to measure starch and water, and a Lindberg/Blue gravity oven was used to treat or cook the samples in the gelatinization time study. All experimental runs were performed in triplicate, with results reported as an average with an error bar equivalent to 1SD (one standard deviation).

### 2.1. Transitional Water Content Determination

The starch/water mixtures prepared for the determination of the transitional water content were analyzed by DSC in increasing concentration from 5% to 60% (starch weight). Samples (total sample weight: 20–25 mg) were loaded into DSC sample pans, sealed, and loaded into a preheated DSC at 40 °C. The samples were allowed to acclimate for at least 30 min, after which they were heated at a rate of 2 °C/min to 130 °C. The resulting DSC curves were used to calculate the gelatinization enthalpies (∆*H*_n_) of the mixtures.

### 2.2. Minimum Cooking/Treatment Temperature Determination

The DSC curves obtained from the transitional water content determination were also used to determine the minimum treatment or gelatinization temperature for WCS in the excess water condition. This minimum temperature is synonymous with the “end temperature” or “conclusion temperature” DSC curve property defined by previous studies [10,12]. Additional details are provided in the Results and Discussion section.

### 2.3. Cooking Time Determination

The thermal treatment or cooking of the starch/water mixtures for the cooking time study was performed in the excess water condition (15% starch by weight) and conducted using the identified minimum cooking temperature. The mixtures were loaded in DSC sample pans, sealed and heat treated in an oven at different times (5, 6, 7, 8, 9, 10, and 15 min). After treatment, the samples were allowed to acclimate at room temperature for 3 h and were re-weighed to ensure that there was no weight loss due to leaks or rupturing of the pans. The samples were then subjected to DSC analysis as previously described to analyze for residual enthalpies (∆*H*_r_).

## 3. Results and Discussion

**Figure 1:**
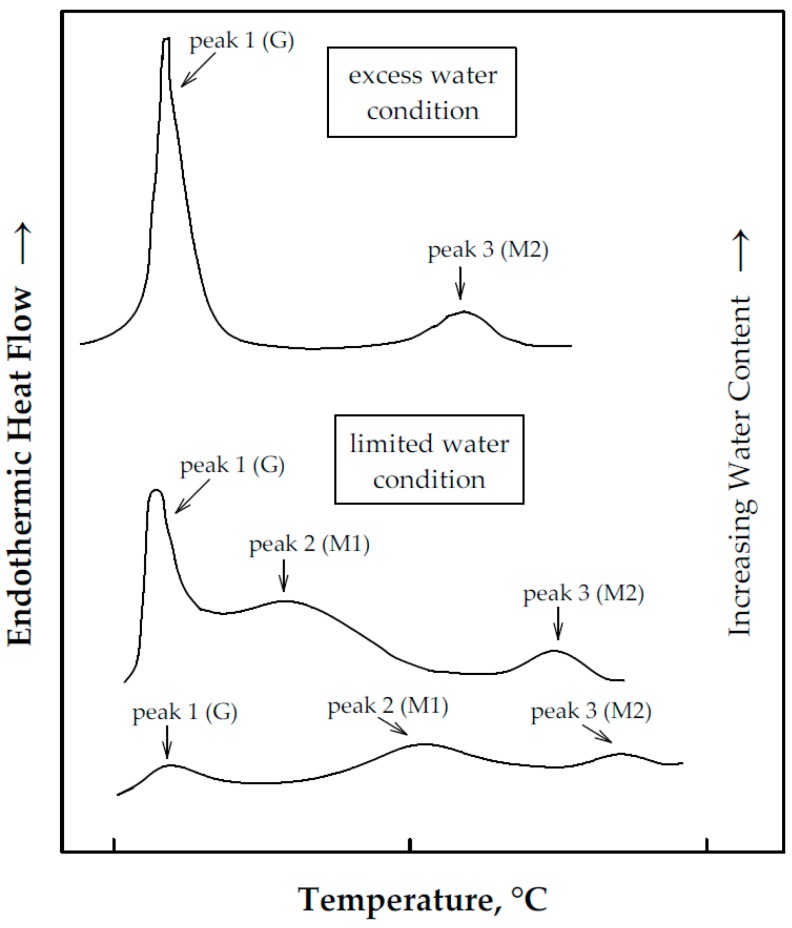
Typical differential scanning calorimetry (DSC) curves of starch/water mixtures at the excess water and limited water conditions. G, M1 and M2 are endotherms. (Redrawn and modified with permission from reference [9]).

Starch gelatinization is characterized by several endotherms which can be identified by DSC. These endotherms appear as peaks on DSC curves, as shown in Figure 1, and are dependent on water content and the type of starch. Peak 1 represents an endotherm that is widely accepted to be related to the gelatinization of amylopectin and has typically been named the G endotherm in previous studies [10,20]. This endotherm does not exist for starches with negligible amylopectin (high amylose starches). Peak 2 represents the non-equilibrium melting of crystallites and is called the M1 endotherm. This endotherm is a characteristic of starch/water mixtures that are in the limited water condition [9,20]. Lastly, Peak 3 (or M2 endotherm) has been attributed to the phase transition of the amylose–lipid complex and is thus uncharacteristic of starches with negligible amount of amylose. This endotherm is also absent when starches are defatted prior to gelatinization [20].

### 3.1. Transitional Water Content

**Figure 2:**
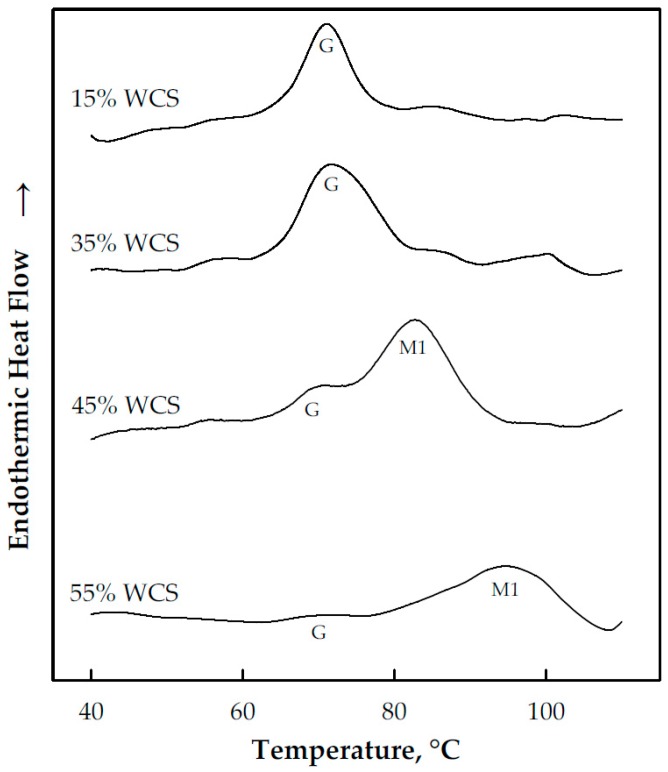
DSC curves of waxy corn starch (WCS) at different levels of water content. The numbers indicate starch content in a wet basis. G, M1 and M2 are endotherms.

The gelatinization of WCS used in this study was characterized by the G and M1 endotherms (see Figure 2), particularly due to the high amylopectin content of the starch. According to Fukuoka et al. [9], the G endotherm can be used as a first-order estimate of ∆*H*_n_, even in the limited water condition due to the small contribution of M1 to the gelatinization enthalpy. This, together with previous observations that the endothermic ∆*H*_n_ can be considered constant and maximum in the excess water region [9,10,11,12], indicates that the G endotherm can be used to identify the transitional water content when the WCS/water mixture shifts from the excess water to limited water conditions.

As shown in Figure 2, the G endotherm peak increased with water content. Consequently, the assumption that the G endotherm approximates the gelatinization enthalpy suggests that ∆*H*_n_ increases with water content as well. Though sound judgement may indicate the inaccuracies of this assumption, it could prove useful in the identification of the transitional water content of waxy starches/water mixtures. This is because M1 is a characteristic of starch/water mixtures in the limited water condition, as previously mentioned.

**Figure 3:**
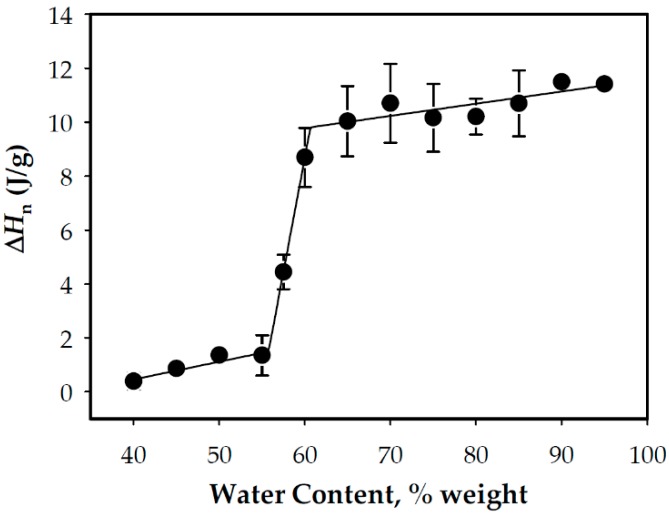
The specific endothermic enthalpy, ∆*H*_n_, based on the G endotherm as a function of the water content in a wet basis.

From the DSC curves, the ∆*H*_n_ of the WCS/water mixtures were estimated, and the results are presented in Figure 3. At low water contents (≤50% weight), low and relatively constant enthalpies were obtained. This was followed by a very steep increase in enthalpies as the water content was increased from 50–60%, which marks the region of overlapping G and M1 endotherms or the region at which M1 separated from the G endotherm. This was followed by an almost constant (and maximum) ∆*H*_n_, as the water content was further increased. The maximum ∆*H*_n_ or ∆*H*_e_ was in the 8–12 J/g range and indicated the region of the excess water condition. In addition, as shown in Figure 2, the transition from the limited water to excess water conditions occurred in the 60–65% water content range, and processing the WCS above this range ensures that the mixture is in the excess water condition where the starch can be fully gelatinized most easily. The obtained ∆*H*_e_ of the WCS used in this study was within the range of waxy starches obtained from different sources, as shown in Table 2.

### 3.2. Cooking Temperature

**Figure 4:**
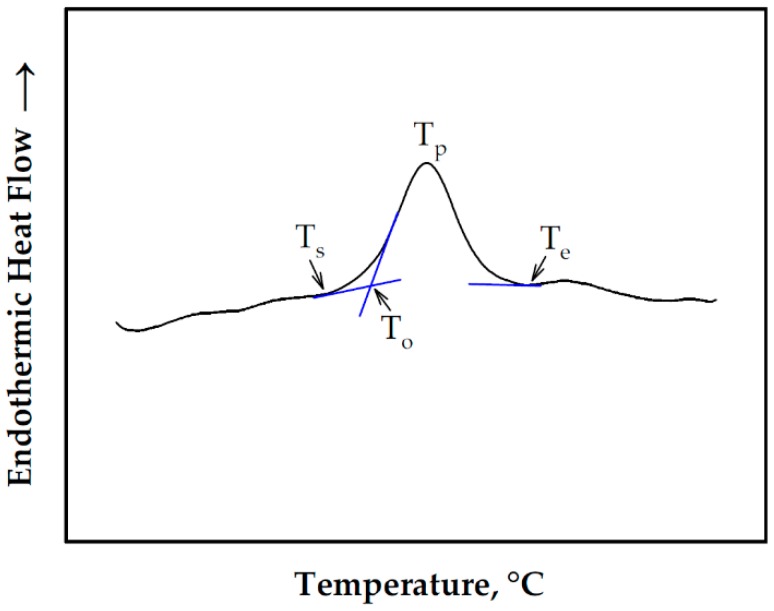
The endotherm characteristics from DSC curves: T_s_—start temperature; T_e_—end temperature; T_o_—onset temperature; T_p_—peak temperature.

In addition to endothermic enthalpies, starch DSC scans can also provide other characteristics pertaining to endotherm peaks, particularly the G and M1. These include start (T_s_), end (T_e_), onset (T_o_), and peak (T_p_) temperatures, as illustrated in Figure 4. The results of this study indicated that T_s_, T_o_ and T_p_ for the G endotherm remained relatively constant across the range of water contents studied (Figure 2). T_e_, on the other hand, was almost unchanged in the excess water condition and increased with decreasing water content in the limited water condition due to the shift of the M1 endotherm towards higher temperature. These results are consistent with the results of other studies such as those of Wang et al. [10], Shiotsubo and Takahashi [12] and Donovan [11]. The peak temperature was in the 70 °C region, which was also consistent with previous studies on WCS [10,20].

**Figure 5:**
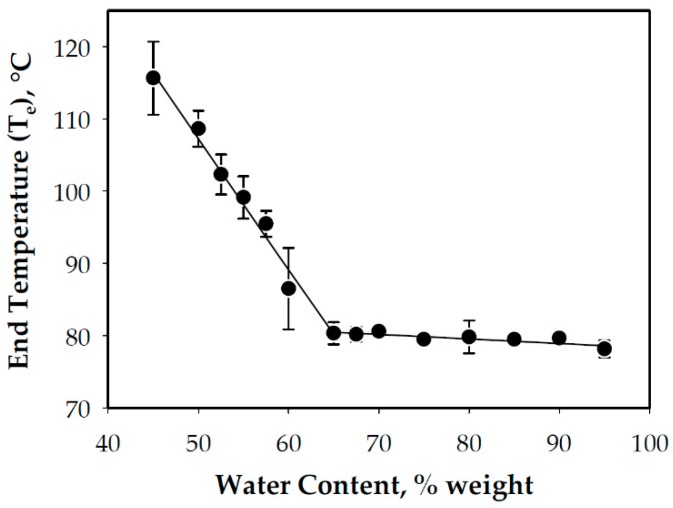
The gelatinization completion temperature as a function of the water content in a wet basis.

For the present study, it was hypothesized that the DSC curves could be used as a basis for the identification of an estimate of the minimum temperature required to fully gelatinize the WCS used in this study. This temperature was the previously defined T_e_ in Figure 4, which for WCS was within the 75–85 °C range in the excess water condition (Figure 5). The study conducted by Ratnayake and Jackson [14] on WCS gelatinization from 35 to 85 °C (at 5 °C increments) indicated that 75 °C is the minimum treatment temperature, which is within the range of T_e_ obtained in this study. This is despite the significance differences between the two studies such as the WCS source and treatment procedure (in-situ or in-DSC pans versus slightly sheared). The nature and source of the starch dictates its thermal characteristics [10,14]. Shearing or agitation during cooking enhances starch swelling, which is exacerbated at elevated temperatures [26]. If the hypothesis of the study could be proven true, this could eliminate tedious works in the identification of a minimum cooking temperature to fully gelatinize other starches. The procedure might not be useful for the elucidation of starch gelatinization mechanisms, but it could prove useful for rapid starch evaluation and process optimization.

### 3.3. Cooking/Gelatinization Time

**Figure 6:**
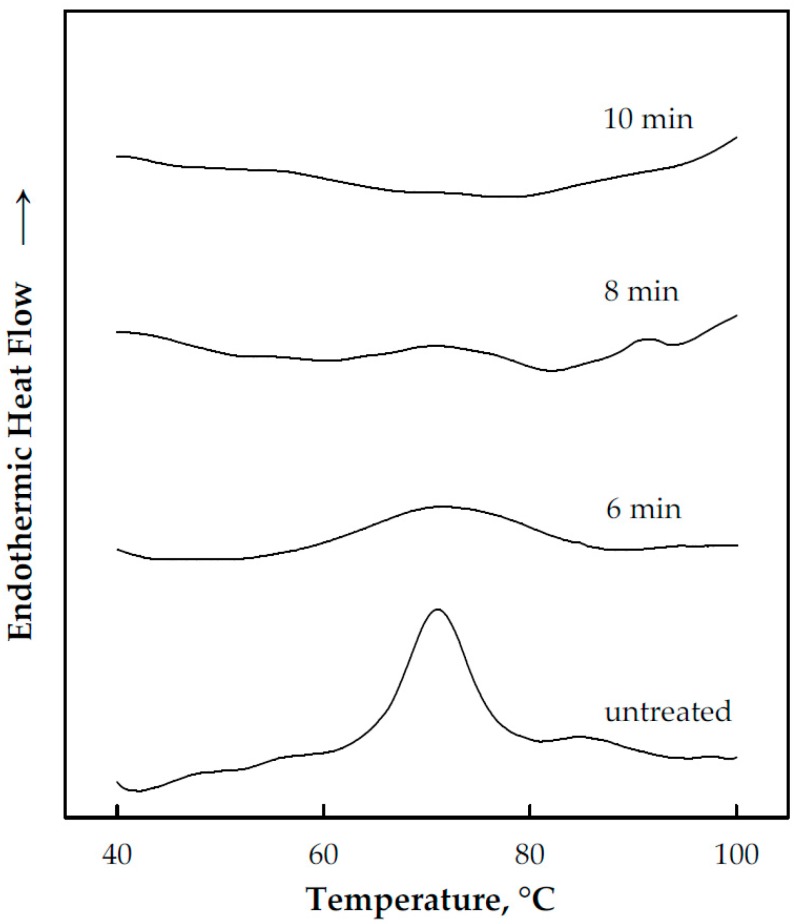
DSC curves at different cooking times at the 85 °C cooking temperature.

**Figure 7:**
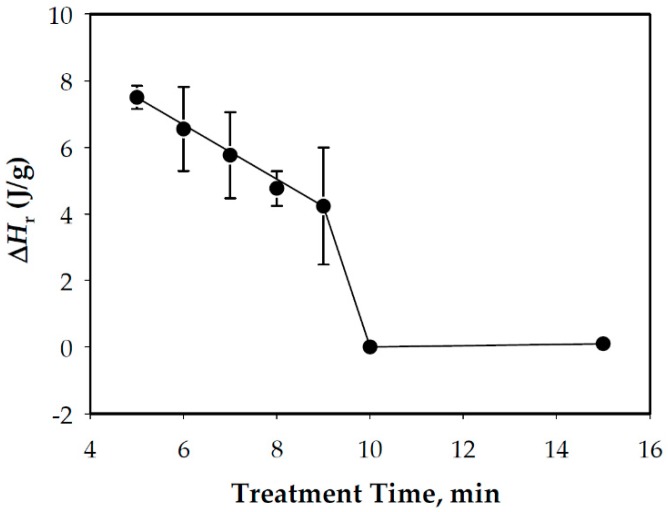
Gelatinization progression as indicated by the disappearance of residual enthalpy, ∆*H*_r_, at the 85 °C treatment temperature.

A WCS/water (contains 85% weight water) mixture in the excess water condition was subjected to thermal treatment at 85 °C, which was previously determined (Section 3.2) as the upper range of minimum temperature for complete WCS gelatinization. Representative DSC curves of the treated samples at different times are presented in Figure 6, which indicate that a cooking time of ~10 min was sufficient enough to fully gelatinize the WCS. This is supported by the calculated ∆*H*_r_ at different treatment times shown in Figure 7.

The obtained cooking time (~10 min) for WCS at the excess water condition and 85 °C was much higher than the one (2–3 min) obtained for rice starch [9]. This was likely due to the differences in the nature and source of the starch which, as previously mentioned, dictate starch’s thermal characteristics. Despite the longer cooking time, the results indicated that WCS could be fully gelatinized at the estimated minimum temperature identified from the DSC curves and proved the previously stated hypothesis.

Since 1984, very few studies [6,13,27] have been conducted on the determination of cooking time for starches. This could be due to the very tedious nature of the experimental determination. For example, in the study conducted by Lund and Wirakartakusumah [6], a starch/water mixture was subjected to various heating/cooking temperatures and several cooking times to determine the temperature and time to completely gelatinize the starch. In contrast, this study has provided a rapid procedure for the estimation of waxy starch gelatinization characteristics; this procedure is as follows: (1.) Determine the transitional water content as outlined in Section 2.1. This step can be optional if the transitional water content is not needed, in which case a water content of ≥90 wt.% can be used for the next steps. (2.) Analyze the waxy starch/water mixture (in the excess water condition) using DSC. Using the DSC curves, the minimum cooking temperature can be estimated, as discussed in Section 3.2. (3.) Subject the waxy starch/water mixture (in the excess water condition) to different cooking times at the minimum cooking temperature followed by a DSC analysis to determine ∆*H*_r_. The cooking time for complete gelatinization is indicated by a negligible ∆*H*_r_ value or the complete disappearance of the G endotherm in the DSC curves.

## 4. Conclusions

Starch is widely used in the food and cosmetic industries as a thickener and stabilizer for emulsified or encapsulated products. Prior to its use in most applications, starch needs to be gelatinized, typically by heat treatment or cooking. Water content, cooking temperature and time dictate the extent by which a given starch can be gelatinized. Starch/water mixtures should ideally be in the excess water condition, subjected to a minimum cooking temperature, and given enough time to fully gelatinize. Thus, for applications that require starches to be fully gelatinized prior to use, the rapid determination or estimation of these parameters is of great interest and was the subject of the present study.

WCS was investigated to determine the gelatinization parameters mentioned above. The determination was conducted using DSC, which provided curves for the calculation of enthalpies (i.e., specific gelatinization enthalpies, ∆*H*_n_, and specific residual gelatinization enthalpies, ∆*H*_r_) and other thermal properties (i.e., T_s_, T_e_, T_o_ and T_p_). The ∆*H*_n_ was utilized for the determination of the excess water condition or, more specifically, the transitional water content where the WCS/water mixture shifted from the limited water to the excess water conditions. During heat treatment, ∆*H*_n_ disappears, leaving ∆*H*_r_ for the identification of a suitable cooking time. During sample scanning, DSC curves provided a temperature profile that could be utilized to estimate the minimum gelatinization temperature (T_e_) required for WCS gelatinization. To the authors’ knowledge, this is the first time that T_e_ was used as an estimate of cooking temperature. For the WCS used in this study, the results showed that the transitional water content was at around 60–65 wt.% water range and a T_e_ value of around 75–85 °C. Furthermore, in the excess water condition, the WCS could be fully gelatinized within 10 min when treated or cooked at 85 °C.

The procedure presented in this study, particularly on the estimation of minimum gelatinization temperature, could provide a useful technique for rapid starch screening or evaluation prior to use. Studies have suggested that for most starches, the minimum gelatinization temperature is within 15 °C of T_e_. This is despite the differences on how the starches are heat treated, some with agitation as opposed to the in situ nature of DSC determination. It is likely that the procedure presented in this study can be extended to starches other than WCS. However, since the thermal properties of starch depends on its source and composition, further experimentation is necessary to claim this with certainty.

## Figures and Tables

**Table 1:** Properties of the waxy corn starch used in this study ^1^.

Parameter	Specification
Moisture	10.0–13.0%
pH	4.5–6.0
Non-Waxy Starch	≤7.0%
Foreign Material	≤10 ppm

^1^ Supplier-provided properties.

**Table 2:** Enthalpy of the gelatinization (∆*H*_e_) of waxy starches.

Starch	∆*H*_e_ (J/g)	Reference
Barley	15.7	[21]
Corn	~16.3	[10]
Corn	17.6	[21]
Corn	8–12	(This study)
Potato	12.36	[22]
Rice	9.6–10.1	[23]
Rice	12.8–14.9	[24]
Wheat	13.3	[25]
